# Effects of destruxin A on hemocytes of the domestic silkworm, *Bombyx mori*

**DOI:** 10.3389/fmicb.2023.1210647

**Published:** 2023-06-02

**Authors:** Fei Yin, Lina Hu, Zhenyu Li, Xiangbing Yang, Paul E. Kendra, Qiongbo Hu

**Affiliations:** ^1^Guangdong Provincial Key Laboratory of High Technology for Plant Protection, Plant Protection Research Institute, Guangdong Academy of Agricultural Sciences, Guangzhou, China; ^2^College of Plant Protection, South China Agricultural University, Guangzhou, China; ^3^Subtropical Horticulture Research Station, USDA-Agricultural Research Service, Miami, FL, United States

**Keywords:** destruxin A, histopathology, hemocytes, target, *Bombyx mori*, entomopathogenic fungus

## Abstract

**Introduction:**

Destruxin A (DA) is a mycotoxin isolated from the entomopathogenic fungus *Metarhizium anisopliae* which has demonstrated inhibitory activity against various insect species. However, the mechanism of inhibition on target sites in insects remains unknown.

**Methods:**

In this research, the dose-response relationship between DA and morphological changes in body tissues and organs of domestic silkworm, *Bombyx mori*, were investigated by histopathological methods to identify the target sites that responded to DA.

**Results and Discussion:**

The results showed that responses of individual tissues and organs varied with DA dosage and treatment time. At low doses (i.e., 0.01μg/g), the hemocytes were the most sensitive to DA with morphological changes apparent at 6 h after treatment. However, the muscle cells, fat body, and Malpighian tubules were unaltered. At higher doses (i.e., > 0.1μg/g), morphological changes were observed in muscle cells, fat body, and Malpighian tubules at 24 h after treatment. The results indicated that DA can be an immunosuppressant by damaging host cells like hemocytes, and at higher doses may potentially impact other physiological processes, including muscle function, metabolism, and excretion. The information presented in the current study will facilitate development of mycopesticides and novel immunosuppressants.

## 1. Introduction

*Metarhizium anisopliae* (Hypocreales: Clavicipitaceae) is an entomopathogenic fungus (EPF) with insecticidal efficacy against various insect pests, with several advantages such as low impact to the environment and minimal development of insecticidal resistance. It has been used successfully as a biological control agent with promising potential as an alternative to conventional synthetic pesticides. However, as with most entomopathogens, pest suppression with EPF is delayed due to the slow infection process on host insects and the time required for EPF establishment in field populations (Batta, 2016). Previous studies on the mechanisms underlying the pathogenicity of EPF reported that several small molecular weight compounds may be involved, and these compounds may contribute to improving the efficacy of EPF as microbial insecticides (Zhao et al., 2016; Wang and Wang, 2017).

Destruxins are non-ribosomal peptides produced in secondary metabolism by EPF, including *M. anisopliae,* that have demonstrated insecticidal attributes. To date, 39 destruxins have been identified from *M. anisopliae*, among which destruxin A (DA) has shown dominant insecticidal activity (Liu and Tzeng, 2012). Reported inhibitory properties of DA include antifeedant, growth regulator, contact toxicity, and ingestion toxicity for more than 20 insect species in the Orthoptera, Lepidoptera, Hemiptera, Coleoptera, and Homoptera (Liu and Tzeng, 2012; Huang et al., 2016). Previous toxicological studies have shown that DA is involved in various physiological processes, functioning as a toxin in the immune system of host insects, including destroying the innate immune system (Pal et al., 2007; Fan et al., 2013), causing changes in cell morphology that lead to organ malfunction in insects (Ruiz-Sanchez et al., 2010; Hunt and Charnley, 2011), rigidifying the muscular system of host insects (Bing et al., 2012), and suppressing proliferation of human cancer cells as a strong anticancer agent (Lafont et al., 2015). The toxicity to insect pests could be due to DA binding to target sites on proteins, such as arginine tRNA synthetase, lamin-C proteins, aminoacyl tRNA synthases, BmTMEM214, and BmSEC13L that resulted in various physiological malfunctions for host insects (Wang et al., 2020, 2021; Yin et al., 2021, 2022). Understanding the mechanisms underlying DA bioactivity is essential for improving the efficacy of EPF as an insect toxicant through molecular manipulation and for isolating strains of *M. anisopliae* with high potential as biopesticides.

Although previous studies on DA-binding proteins have been conducted, the pathway of EPF infection in insects, particularly the role of DA in disrupting physiological processes, remains unknown. The susceptibility and sensitivity of host tissues and organs to DA and the process of pathogenicity of DA infection in insects is still unclear. According to previous research results, it is speculated that DA may act on different insect tissues or organs at varied concentrations, but the most susceptible tissues to DA are those involved in immune system. To better understand the functional characteristics of DA in the pathogenic process, the domestic silkworm, *Bombyx mori* L. (Lepidoptera: Bombycidae), was used as a host insect. In this study, DA was injected into larval *B. mori* to determine the effect on host organs and tissues, including the excretory organ, fat body, Malpighian tubules, midgut, and hemocytes. The organs and tissues were examined via paraffin sectioning, transmission electron microscopy, and scanning electron microscopy to document the morphological changes.

## 2. Materials and methods

### 2.1. Chemicals

DA (95%) was purchased from Sigma-Aldrich (Darmstadt, Germany). Chlorpyrifos (analytical standard, 99%) and rotenone were purchased from Shanghai Aladdin Biochemical Technology Co., Ltd. (Shanghai, China). Prior to conducting the experiments, DA was dissolved in dimethyl sulfoxide (DMSO; Sigma-Aldrich) to make a stock solution of 10 mg/mL, which was stored in aliquots in a −80°C ultralow freezer (DW-86L338J, Haier, China).

### 2.2. Insects

The domestic silkworm, *Bombyx mori*, was obtained from the Institute of Sericulture and Agricultural Product Processing, Guangdong Academy of Agricultural Sciences, Guangzhou, China. The *B. mori* colony was maintained on leaves of white mulberry, *Morus alba* (Rosales: Moraceae), in an insectary at 26.0 ± 1.0°C, 80.0 ± 2.0% relative humidity, and photoperiod of 16:8 h (light:dark). To obtain cohorts of known age, eggs (≤3 days old) were collected initially, then the larvae that hatched within 24 h were transferred to mulberry leaves for rearing.

The 2nd and 5th instar larvae of *B. mori* were used for experiments. DA, rotenone, and chlorpyrifos were injected into the larval hemolymph of the third proleg using a microinjection pump (Nanoliter 2010, World Precision Instruments, Germany). After injections, the larvae were kept at 26.0 ± 1.0°C. Chemical doses evaluated are indicated in sections 2.3–2.5 below. Thirty larvae were injected at each dose rate for each chemical, and the larvae without any discharge of chemical post-injection were selected for experiments. Non-injected larvae were used as untreated controls.

### 2.3. Paraffin sectioning of organs and tissues after chemical injection

Paraffin sectioning was used to examine the effects of DA, rotenone, and chlorpyrifos on organs and tissues of *B. mori* larvae. The 2nd instar larvae were injected with the three chemicals; for each chemical, three concentrations were injected and the dosage was based on a weight ratio of chemical to larval body weight (μg _chemical_/g _lava_). For DA, the dose rates injected were 4.0, 2.0, and 0.1 μg/g; for chlorpyrifos, dose rates were 6.0, 2.0, and 0.1 μg/g, and for rotenone, dose rates were 8.0, 4.0, and 0.1 μg/g. The treated larvae were examined at 1, 2, 4, 6, and 12 h after injection.

To prepare the organ and tissue samples for paraffin sectioning, a series of steps including dehydration, waxing and dewaxing, and dyeing were performed, following the protocols of Vinha et al. (2021). Specifically, the treated *B. mori* for each dose were first collected and placed in 4% paraformaldehyde at 4°C in a refrigerator for 24 h. Then each sample was processed using this sequential procedure: dehydration in 75% ethanol for 4 h, 85% ethanol for 2 h, 90% ethanol for 2 h, 95% ethanol for 1 h, 30 min in anhydrous ethanol twice, 8 min in ethanol and benzene, and 8 min in xylene twice. After dehydration, larvae were removed for the waxing and dewaxing process. After wax dipping for 3 h, the samples were placed into the embedding machine for tissue embedding. The wax block containing organ or tissue samples was then cut into thin slices, 4 μm in thickness, by a paraffin slicer. The cut slices were placed on the spreading machine to flatten them, followed by careful placement of the tissue samples on slides for baking at 60°C for 8 h. The slides were then removed from the oven for dewaxing and dyeing. To dewax, the slices were soaked as follows: xylene (I) for 20 min, xylene (II) for 20 min, anhydrous ethanol (I) for 10 min, anhydrous ethanol (II) for 10 min, 95% ethanol for 5 min, 90% ethanol for 5 min, 80% ethanol for 5 min, 70% ethanol for 5 min, and rinsed with distilled water. To dye, the paraffin sections were soaked in Harris hematoxylin dye (Wuhan Goodbio Technology Co., Ltd.) for 3–8 min, then rinsed with distilled water, soaked again in 1% hydrochloric acid ethanol to differentiate the dye for 5 s and rinsed with distilled water, then treated with 0.6% ammonia water and washed with flowing water. The dyed slices were placed in eosin staining solution for 1–3 min. The stained slices were then dehydrated in a series of solutions: 95% ethanol for 5 min twice, anhydrous ethanol for 5 min twice, xylene for 5 min twice, then removed and dried in preparation for imaging. The slices were then observed under an optical microscope (Nikon Eclipse CI, Nikon, Japan) and photographs were taken and analyzed.

### 2.4. Ultrastructural examination of fat body and Malpighian tubules

Second instar larvae were injected with DA at doses of 1.0, 0.1, and 0.01 μg/g for 6 or 24 h, and then observed for ultrastructural changes in fat body and Malpighian tubules. After treatment, larvae were washed with distilled water, dried with paper towels, sterilized with 75% ethanol and air dried. Sample preparation consisted of a series of steps including dissection, fixation, dehydration, and staining. Specifically, larvae in each dose treatment were placed in saline solution (0.1 M NaCl, 0.1 M KH2PO4, 0.1 M Na2HPO4) and dissected quickly under an anatomical microscope (SZ51-ILST-SET, Olympus, Japan). The Malpighian tubules and fat body were carefully removed using forceps or scalpel and immediately soaked in fixation solution (2.5% glutaraldehyde at PH7.2 + 20% paraformaldehyde) and transferred to a refrigerator at 4°C. After 12 h, the fixed samples were rinsed with phosphate buffer (0.1 mol/L, pH 7.2) 6 times, with 10 min for each rinse. The samples were fixed again using 1% Osmic acid solution (OsO4) for 1 h at 4°C, then rinsed with phosphate buffer (6 times, 10 min each), and dehydrated with 30, 50, and 70% ethanol, respectively. The samples were dehydrated further with 80% ethanol (15 min), 90% ethanol (20 min), and 100% ethanol (30 min). The dehydrated samples were permeated at 18°C with ethanol:LR White resin (3:1) for 2 h, ethanol:LR White (1:1) for 4 h, ethanol:LR White (1:3) for 12 h, and pure LR White for 24 h, repeated 2 times. Finally, the sample was embedded in pure LR White in the capsule, and the embedded sample was put in the oven at 30°C for 24 h and then 60°C for 48 h to ensure polymerization. The sample pieces of the polymerized capsules were semi-thin sliced with a Leica EM UC7 slicer. The semi-thin section was stained with 1% toluidine blue and observed under a microscope (Nikon Eclipse CI, Nikon, Japan). Under the microscope, samples were then cut into ultra-thin slices with 80 nm thickness. The ultra-thin slices were double stained with 2% uranyl acetate (10 min) and 4% lead citrate (8 min). The stained specimens were observed and photographed with a JEM-1200EX tungsten filament transmission electron microscope (80 kV) for image analysis (Tian et al., 2009).

### 2.5. Scanning electron microscopy (SEM) of hemocytes

The response of hemocytes to DA treatments was examined by scanning electron microscopy (SEM) following previously published methods with slight modification (Fan et al., 2013). Fifth instar larvae were injected with DA at dose rates of 0.01, 0.10, and 1.00 μg/g for 6 or 24 h, then washed with distilled water and disinfected with 75% alcohol. The abdominal foot of the silkworm was cut with disinfected surgical scissors, and the hemolymph was collected directly into precooled 2.5% glutaraldehyde fixation solution (3 mL). After 24 h, the samples were centrifuged for 10 min at 5,000 r/min and the supernatant was removed. The remaining hemocytes were rinsed with phosphate buffer solution (PBS) for 20 min, repeated for 4 times, then dehydrated with 30, 50, and 70% ethanol consecutively for 10 min at each concentration. The samples were dripped onto a clean copper table and put into an automatic CO_2_ critical point dryer for 1 h. The samples were then mounted on stubs for coating (sputter coater) and analyzed by SU8020 SEM (Hitachi Limited, Japan) for data documentation.

## 3. Results

### 3.1. Paraffin sectioning of tissues and organs after DA treatment

**Figure 1:**
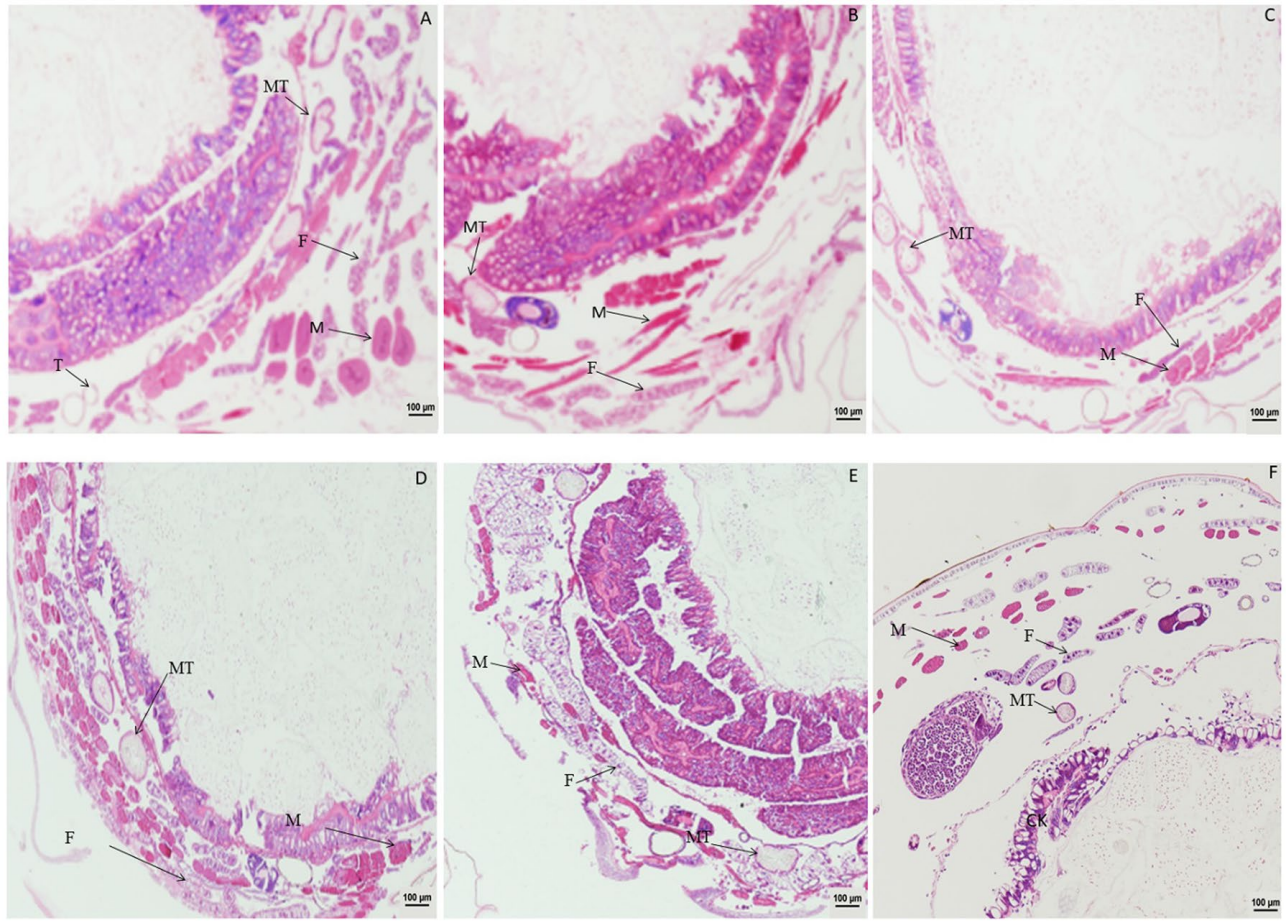
Paraffin sectioning of tissue of *Bombyx mori* larvae treated with 4 μg/g DA. MT, Malpighian tubule; S, Silk gland; F, Fat; M, Muscle; T, Trachea. **(A)** At 1 h after the treatment, the muscle cells were enlarged. **(B)** At 2 h after the treatment, the muscle cells became elongated. **(C)** At 4 h after treatment, the muscle cells were expanded and elongated than those in control. **(D)** At 12 h after treatment, the wall of Malpighian tubules became thinner except for those of the expanded and elongated muscle cells. **(E)** At 24 h after treatment, the vacuolation of fat body became more significant. **(F)** Untreated control.

Significant changes in morphology of muscle cells were observed after treatment with DA (Figure 1). When larvae were treated with DA at 4.0 μg/g, muscle cells were enlarged compared to those in the control at 1 h after treatment, and became elongated at 2 h after treatment. At 4 h after treatment, the muscle cells were expanded and elongated significantly compared to those in the control. At 12 h after treatment, the walls of Malpighian tubules became thinner except for those in muscle cells. The vacuolation of fat body became more significant, and muscle cell expansion increased with the time after treatment.

**Figure 2:**
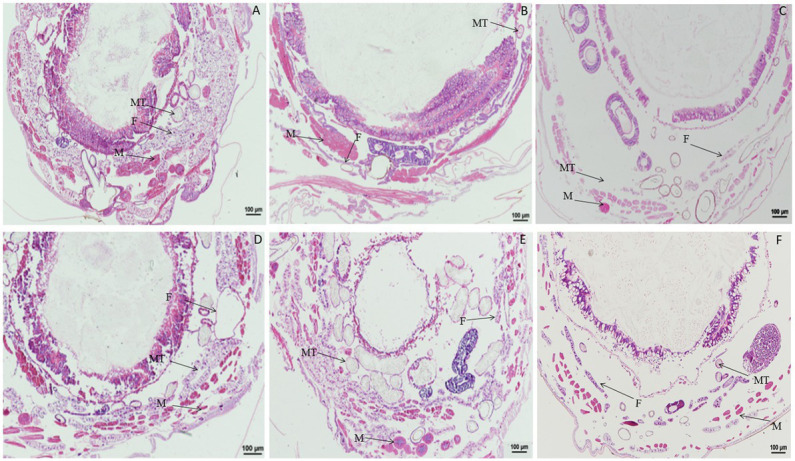
Paraffin sectioning of *Bombyx mori* larvae treated with 2 μg/g DA. MT, Malpighian tubule; S, Silk gland; F, Fat body; M, Muscle; T, Trachea. **(A)** At 1 h after the treatment, the muscle cells were enlarged that those in control. **(B)** At 2 h after the treatment, the muscle cells became elongated and expanded, the vacuolation of fat body became more significant. **(C)** At 4 h after the treatment, the muscle cells were expanded and the vacuolation of fat body became more significant. **(D)** At 12 h after the treatment, the muscle cells were expanded and the fat bodies were vacuolated. **(E)** At 24 h after the treatment, the muscle cells were expanded and the vacuolation of fat body became more significant. **(F)** Untreated control.

**Figure 3:**
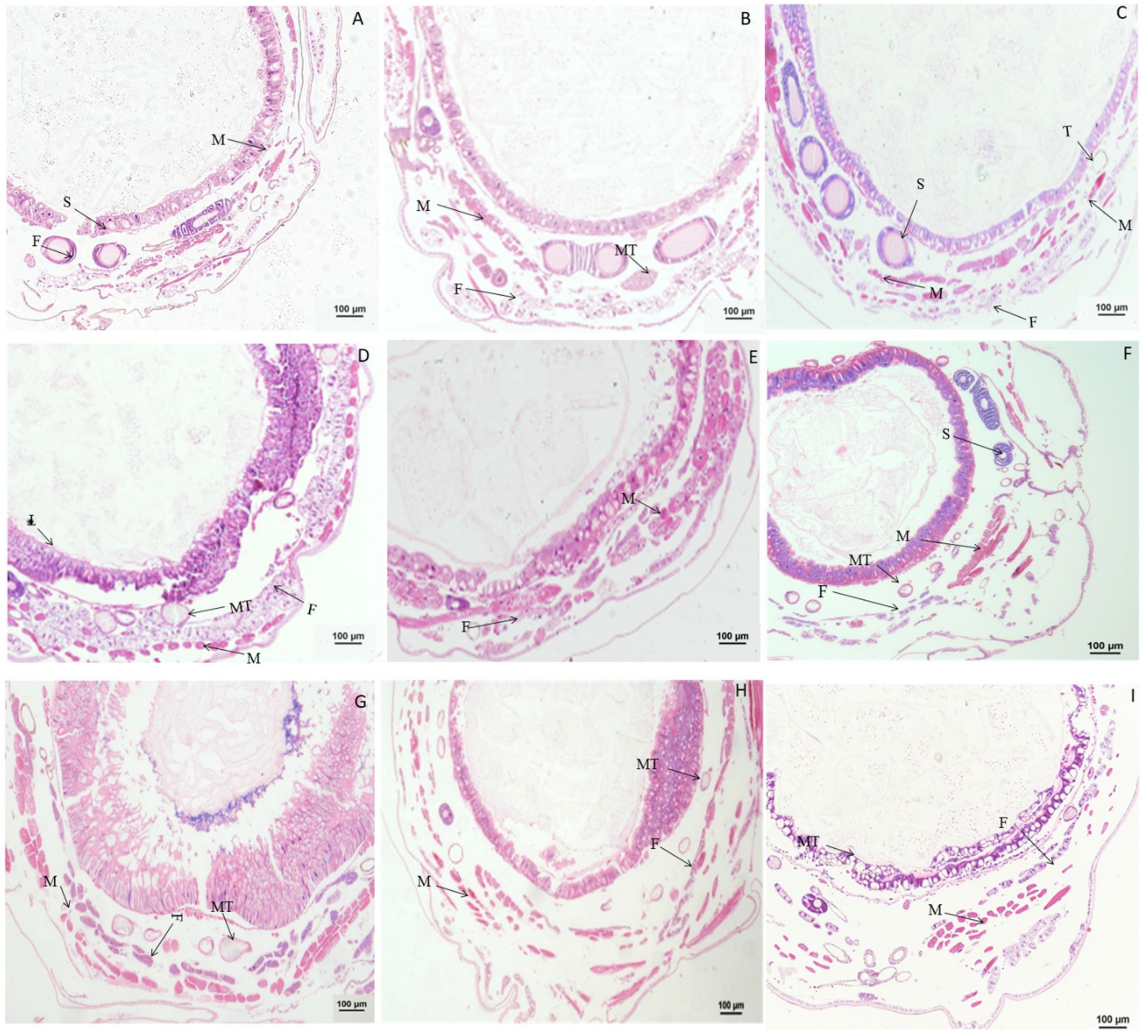
Paraffin sectioning of *Bombyx mori* larvae treated with 0.1 μg/g DA. MT, Malpighian tubule; S, Silk gland; F, Fat; M, Muscle; T, Trachea. **(A–H)** The *B. mori* larvae were treated with DA at 0.5/1/2/6/24/48/72/96 h, respectively. **(I)** Untreated control. When larvae were treated with 0.1 μg/g DA, no significant changes were observed at all times after 0.1 μg/g DA treatment for the muscle cells, fat body, and Malpighian tubules.

When larvae were treated with DA at 2.0 μg/g, compared with the control, the muscle cells were enlarged at 1 h after treatment, and fat body was significantly vacuolated at 2 or 6 h after treatment. No change was observed in the Malpighian tubules (Figure 2). When larvae were treated with DA at 0.1 μg/g, no significant changes were observed regardless of treatment duration for the muscle cells, fat body, and Malpighian tubules of *B. mori* (Figure 3).

### 3.2. Paraffin sectioning of tissues and organs after treatment with chlorpyrifos

**Figure 4:**
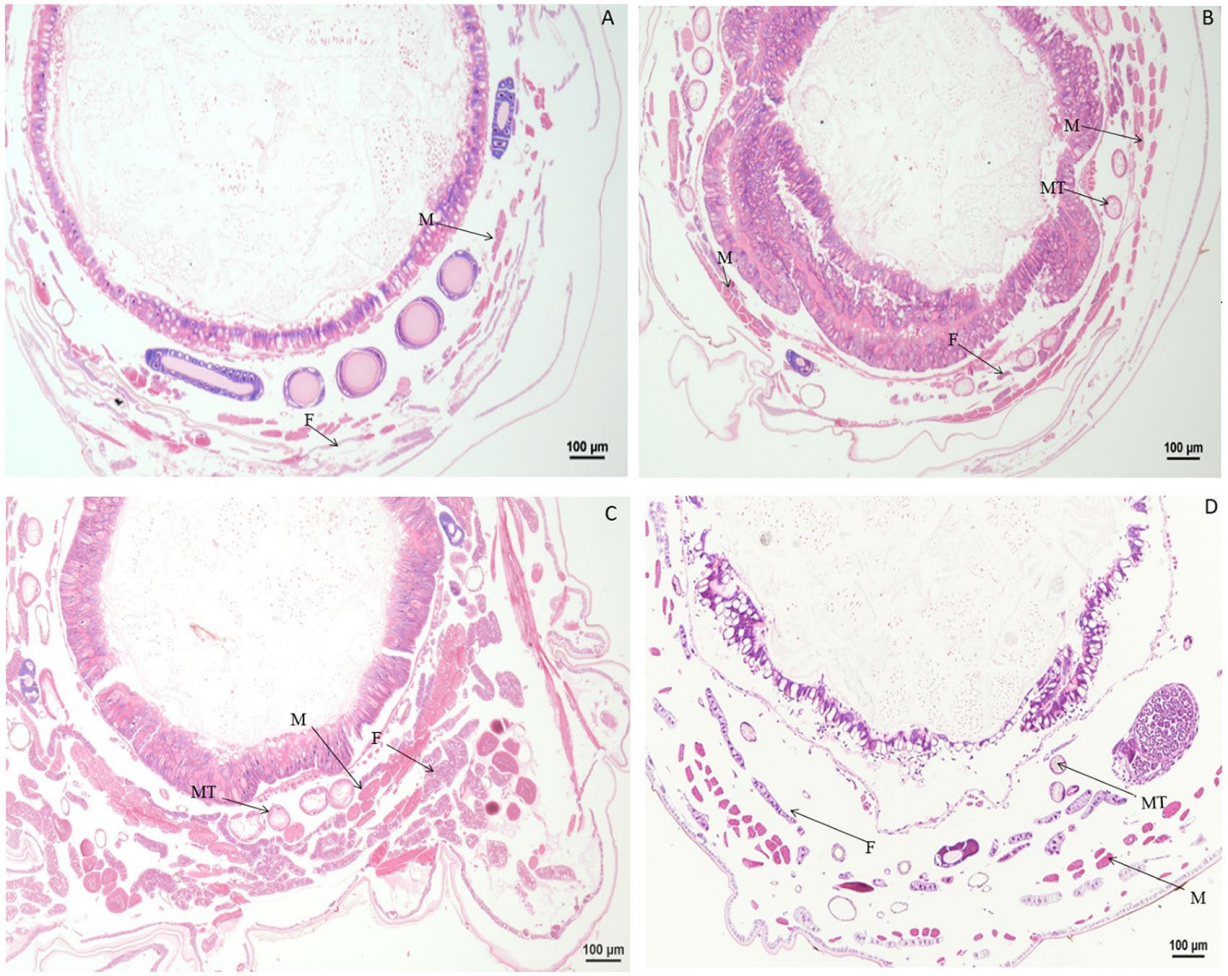
Paraffin sectioning of tissue of *Bombyx mori* larvae treated with 1 μg/g chlorpyrifos. MT, Malpighian tubule; S, Silk gland; F, Fat body; M, Muscle; T, Trachea. **(A)** At 1 h after the treatment, the muscle cells were slightly elongated. **(B)** At 2 h after the treatment, the muscle cells were slightly elongated. **(C)** At 4 h after the treatment, the muscle cells were slightly elongated. The vacuolization of fat body was aggravated, and the muscle cells were further enlarged. **(D)** Untreated control.

Significant changes in morphology were observed in tissues and organs of *B. mori* after treatment with chlorpyrifos. When larvae were treated with chlorpyrifos at 1.0 μg/g for 1 or 2 h, no morphological changes were found in the fat body as compared with the untreated control, but the muscle cells were slightly elongated after treatment. When larvae were treated with 1.0 μg/g for 4 h, the fat body was vacuolated, and the muscle cells were further enlarged (Figure 4).

**Figure 5:**
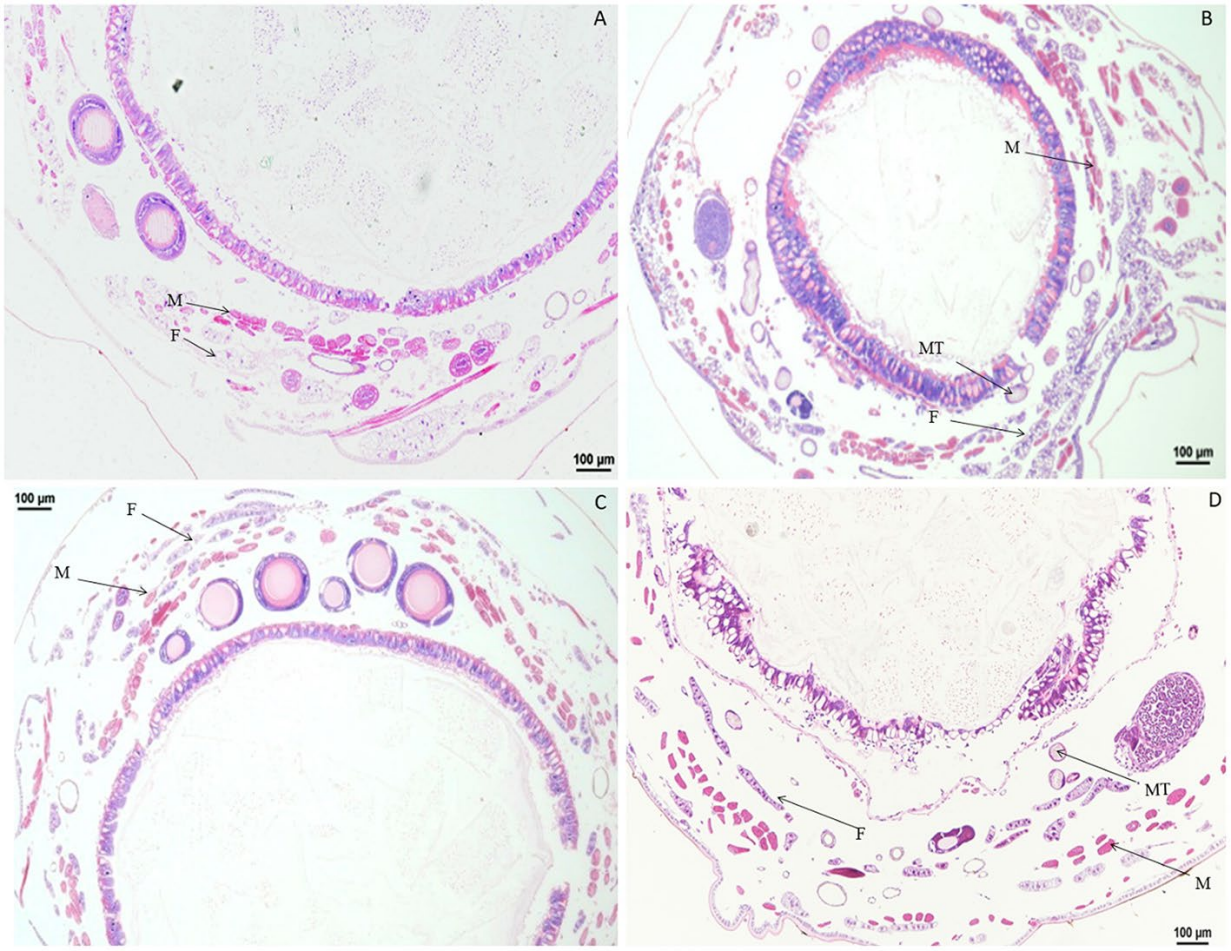
Paraffin sectioning of tissue of *Bombyx mori* larvae treated with 0.1 μg/g chlorpyrifos. MT, Malpighian tubule; F, Fat body; M, Muscle. **(A)** At 12 h after the treatment, the vacuolation of fat body were intensified, and the muscle cells remain elongated or enlarged. **(B)** At 24 h after the treatment, the vacuole ratio of fat body decreased. **(C)** At 72 h after the treatment, the muscle cells and fat body returned to normal as those in the control treatment. **(D)** Untreated control.

Compared with the control, no morphological changes were found in the muscle cells, Malpighian tubules, and fat body of larvae treated with 0.1 μg/g chlorpyrifos at 30 min and 1 h after treatment. However, at 4 h after treatment, the fat body began to vacuolate and the muscle cells were elongated. At 12 h after treatment, the vacuolation of fat body was intensified, and the muscle cells remain elongated or enlarged, but the vacuole ratio of fat body decreased at 24 h after treatment. At 72 h after treatment, the muscle cells and fat body returned to normal and were similar in appearance to the control treatment (Figure 5).

### 3.3. Paraffin sectioning of tissues and organs after treatment with rotenone

**Figure 6:**
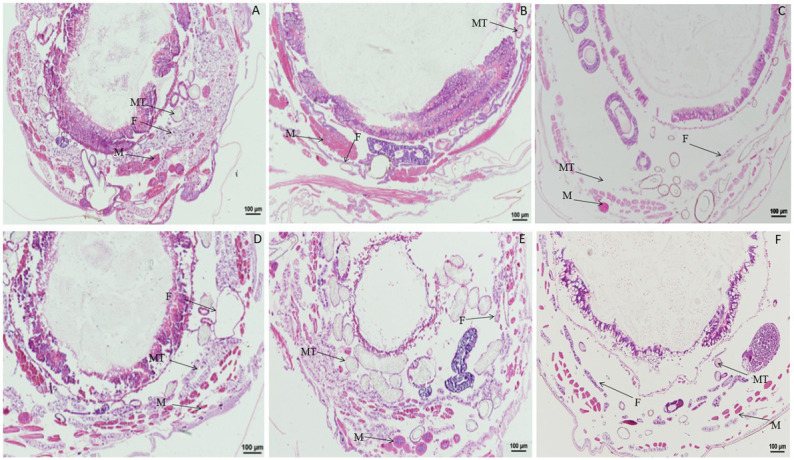
Paraffin sectioning of tissue of *Bombyx mori* larvae treated with 8 μg/g rotenone. MT, Malpighian tubule; F, Fat body; M, Muscle. **(A)** At 1 h after the treatment, the fat body was significantly vacuolated, the muscle cells and the Malpighian tubules were enlarged. **(B)** At 2 h after the treatment, the muscle cells were elongated, and the fat body decreased, but no changes were observed in the Malpighian tubules. **(C)** At 4 h after the treatment, the muscle cells and the Malpighian tubules were enlarged except the fat body that remained unchanged. **(D)** At 12 h after the treatment, the muscle cells were enlarged, the fat body was vacuolated and the Malpighian tubules was deformed, when compared with those in control. **(E)** At 24 h after the treatment, the Peritrophic matrix began to dissociate, the muscle cells shrunk in size, the fat body was expanded, and the Malpighian tubules were deformed. **(F)** Untreated control.

Significant morphological changes in tissues and organs of *B. mori* were observed after treatment with rotenone. When larvae were treated with rotenone at a dose of 8.0 μg/g for 1 h, the fat body was significantly vacuolated, and both the muscle cells and the Malpighian tubules were enlarged. At 2 h after treatment, the muscle cells were elongated and the fat body decreased, but no changes were found in the Malpighian tubules, when compared with larvae in the control treatment. At 4 h after treatment, the muscle cells and the Malpighian tubules were enlarged, but the fat body remained unchanged, as compared with those in the control treatment. At 12 h after treatment, the muscle cells were enlarged, the fat body was vacuolated and the Malpighian tubules were deformed, when compared with those in the control. At 24 h after treatment, the peritrophic membrane began to dissociate, the muscle cells shrunk in size, the fat body was expanded and the Malpighian tubules were deformed, compared to those in the control treatment (Figure 6).

**Figure 7:**
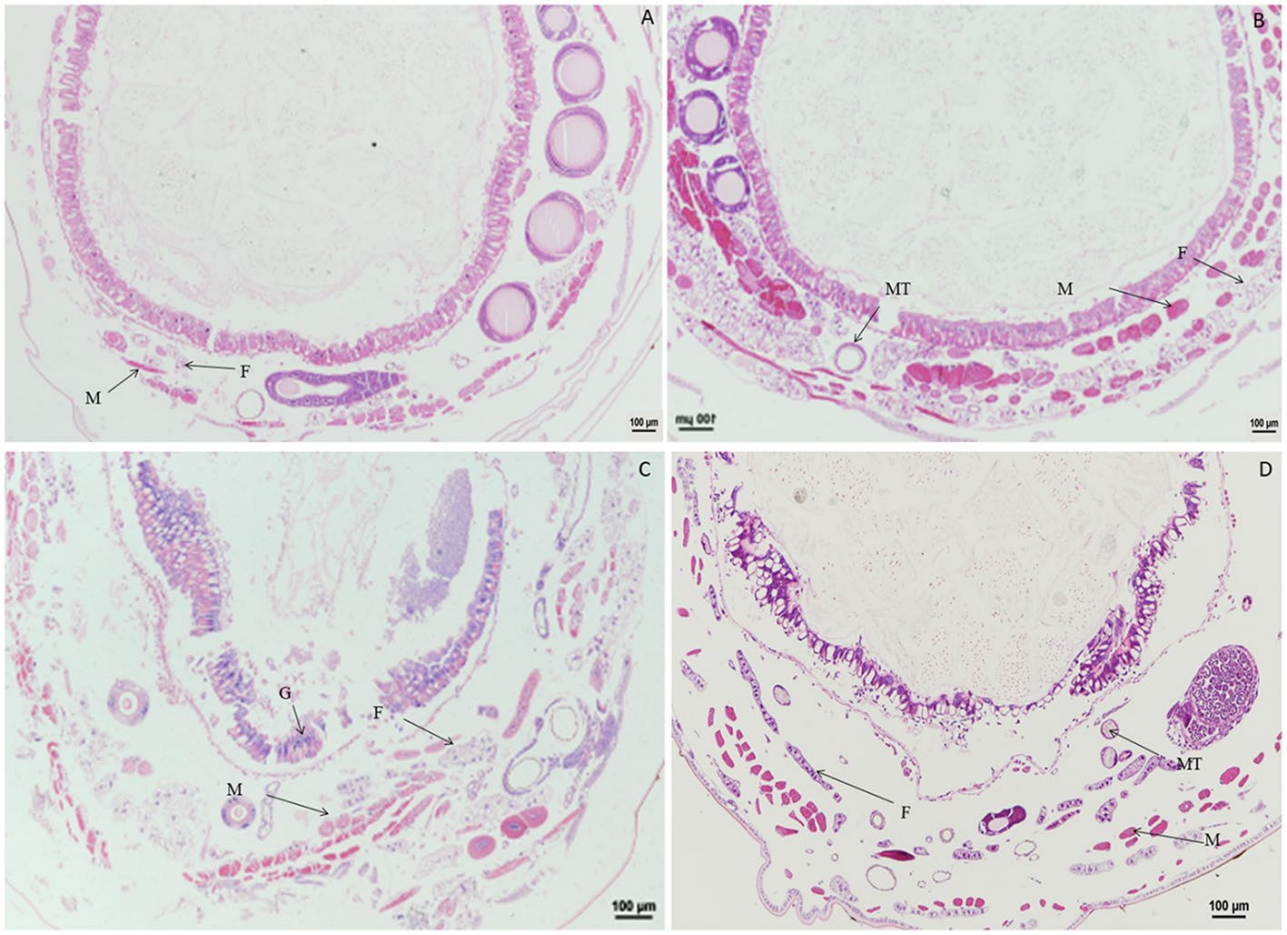
Paraffin sectioning of tissue of *Bombyx mori* larvae treated with 4 μg/g rotenone. MT, Malpighian tubule; F, Fat body; M, Muscle; G, Gut wall. **(A)** At 12 h after treatment, the fat body was vacuolated and the muscle cells were elongated. **(B)** At 24 h after treatment, the vacuolation of fat body was aggravated, the muscle cells were enlarged and the Malpighian tubules remained unchanged. **(C)** At 36 h after treatment, the gut wall was dissociated, the fat body was vacuolated, and the muscle cells were enlarged or elongated. **(D)** Untreated control.

When larvae were treated with rotenone at a dose of 4.0 μg/g for 1.0, 2.0, and 4.0 h, the muscle cells were expanded, but the fat body and Malpighian tubules remained unchanged. At 12 h after treatment, the fat body was vacuolated and the muscle cells were elongated. At 24 h after treatment, the vacuolation of fat body was more extensive, the muscle cells were enlarged and the Malpighian tubules remained unchanged. At 36 or 48 h after treatment, the gut wall was dissociated, the fat body was vacuolated, and the muscle cells were enlarged or elongated (Figure 7).

**Figure 8:**
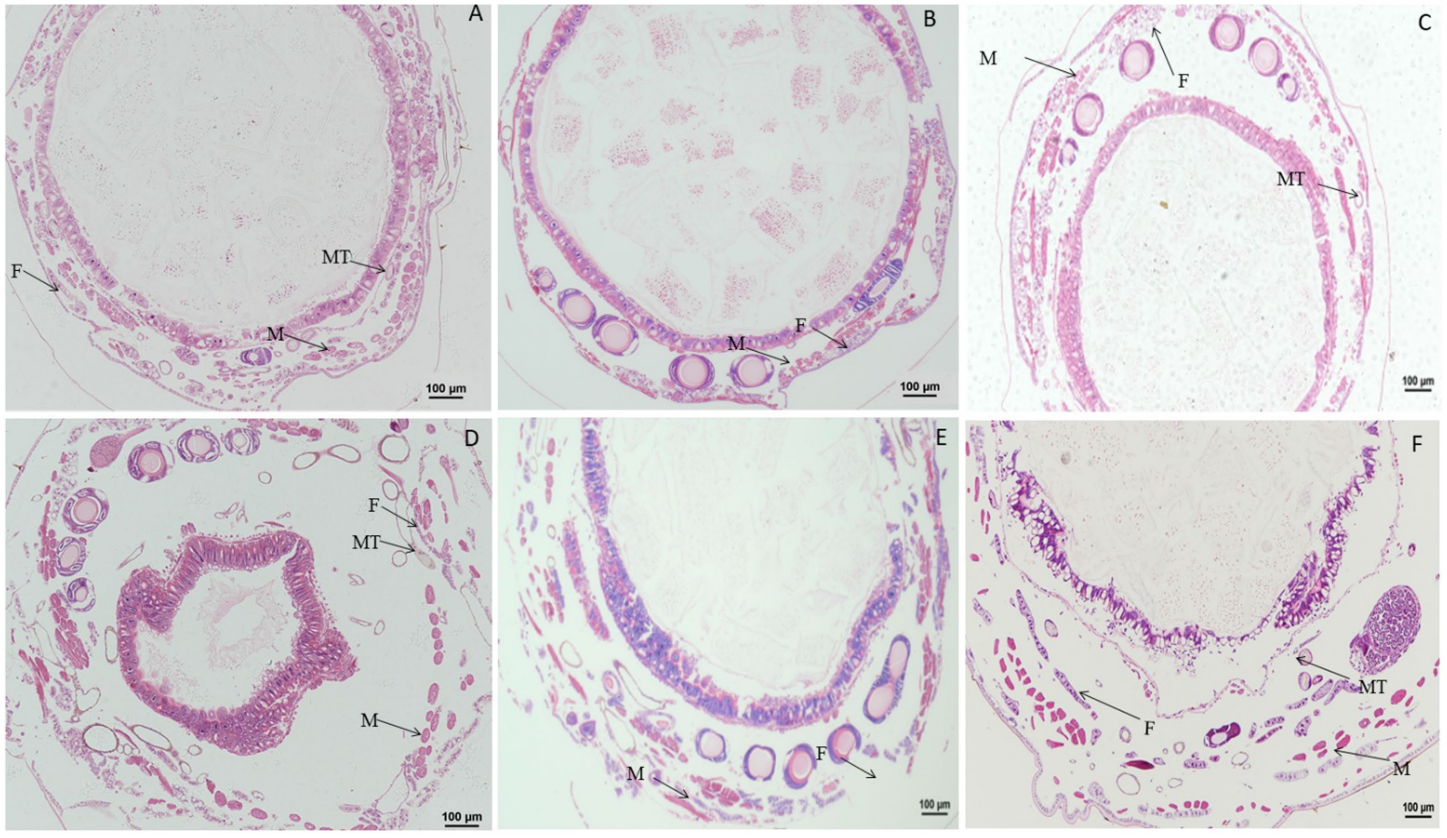
Paraffin sectioning of tissue of *Bombyx mori* larvae treated with 0.1 μg/g rotenone. MT, Malpighian tubule; F, Fat; M, Muscle. **(A)** At 30 min after treatment, the muscle cells were enlarged and the fat body was vacuolated. **(B)** At 1 h after treatment, the vacuolation of fat body was aggravated. **(C)** At 24 h after treatment, the vacuolation of fat body was aggravated and the muscle cells were elongated, the wall of Malpighian tubules became thinner. **(D)** At 48 h after treatment, the muscle cells were elongated and expanded, the fat body returned to normal and the wall of Malpighian tubules became thinner. **(E)** At 72 h after treatment, the fat body and muscle cells resumed to normal. **(F)** Untreated control.

When larvae were treated with rotenone at a dose of 0.1 μg/g for 30 min, the muscle cells were enlarged and the fat body was vacuolated. At 1 h after treatment, the vacuolation of fat body increased. At 24 h after treatment, the vacuolation of fat body was more extensive and the muscle cells were elongated. At 48 h after treatment, the muscle cells were elongated and expanded, the fat body appeared normal and the wall of Malpighian tubules became thinner. At 72 h after treatment, both the fat body and muscle cells were morphologically no different than those in the control (Figure 8).

### 3.4. Effect of DA on the ultrastructure of tissues and organs

**Figure 9:**
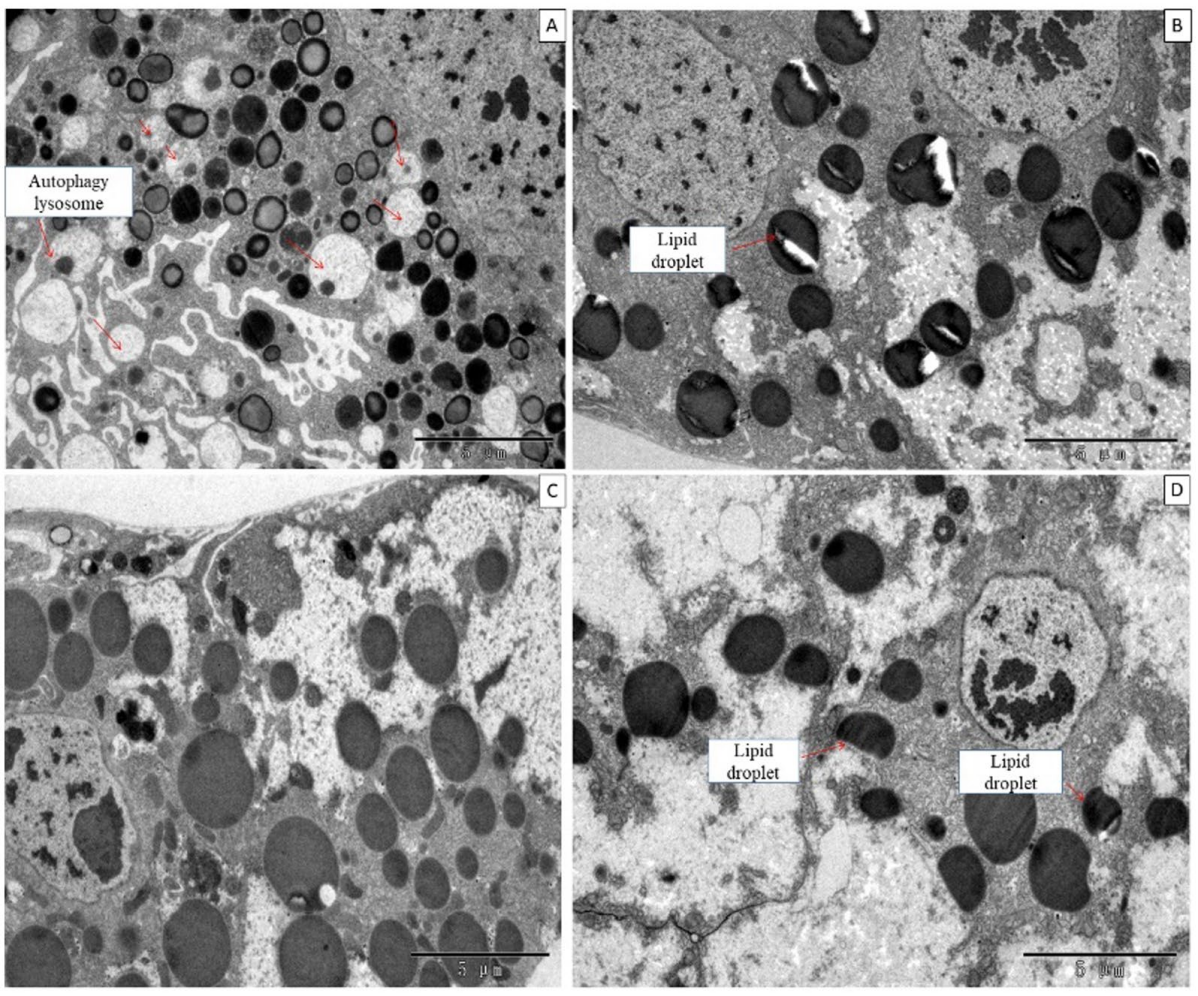
Ultrastructure of the fat body of *Bombyx mori* larvae treated with DA for 24 h. **(A)** The fat droplets became semicircular or deformed at 24 h after treatment at 0.01 μg/g of DA. **(B)** The fat droplet became incomplete or split at 24 h after treatment at 0.1 μg/g of DA. **(C)** Untreated control. **(D)** The fat droplets became semicircular or deformed at 24 h after treatment at 1 μg/g DA.

When larvae were treated with DA at 0.01, 0.10, or 1.0 μg/g for 6 h, the fat body showed no morphological changes, but the number of fat body cells and lysosomes were increased at 0.01 μg/g DA at 24 h after treatment. When larvae were treated with DA at 0.1 μg/g for 24 h, the fat droplets became incomplete or split as compared with those in the control. When larvae were treated with DA at 1.0 μg/g for 24 h, the fat droplets became semicircular or deformed, as shown in Figure 9.

**Figure 10:**
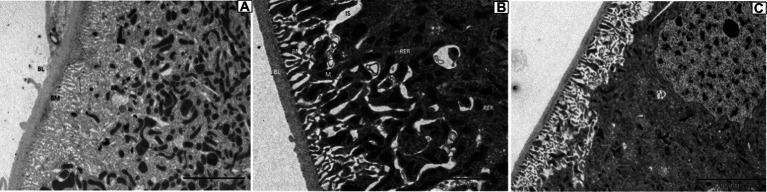
Ultrastructure of the Malpighian tubules of *Bombyx mori* larvae treated at 24 h after treatment. **(A)** 1 μg/g of DA treatment. **(B)** 0.1 μg/g of DA treatment. **(C)** Untreated control. BL, basal lamina; BM, cell basal membrane basal plasma membrane; IS, intercellular space; M, mitochondrial mitochondrion; N, nucleolus; RER, rough endoplasmic reticulum.

For Malpighian tubules, no significant changes were observed in larvae treated with DA at 1.0 and 0.1 μg/g for 24 h (Figure 10).

**Figure 11:**
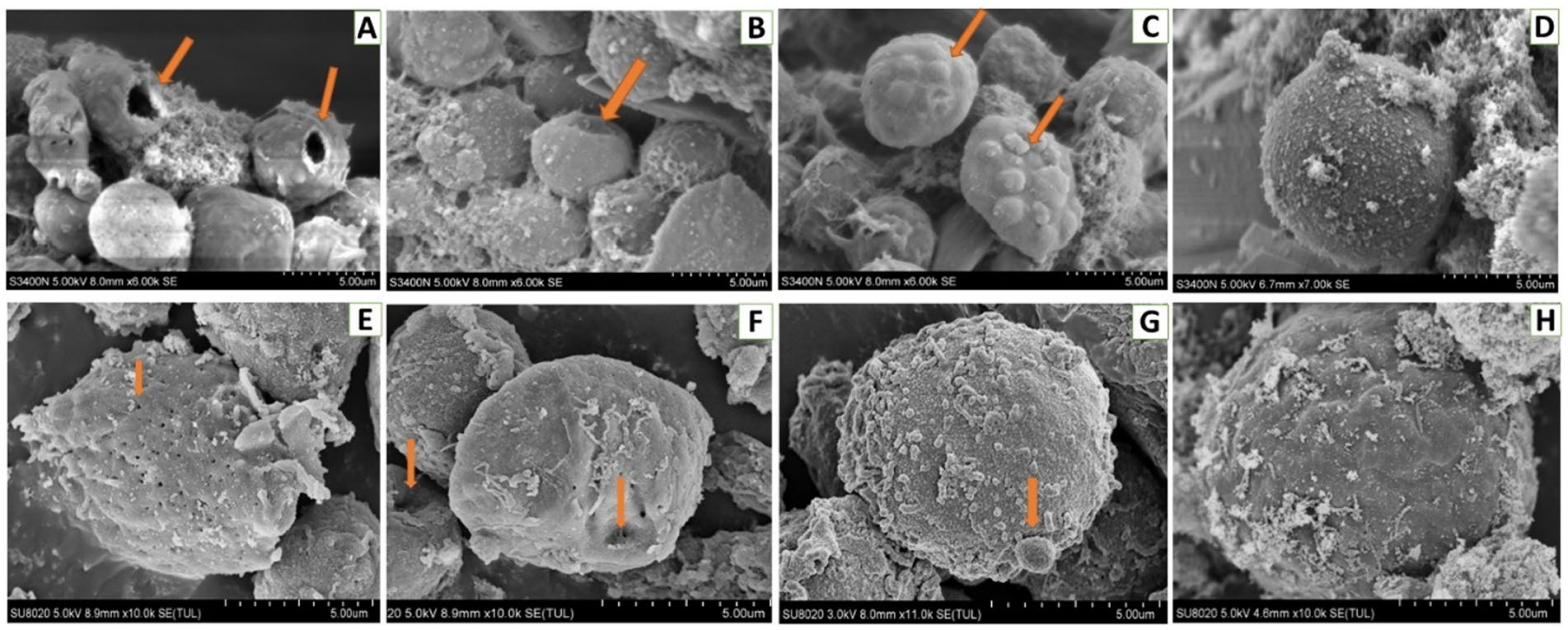
Scanning electron microscopic analysis of hemocytes of *Bombyx Mori* larvae treated with 0.01 μg/g of DA. **(A)** Perforation, at 6 h after treatment. **(B)** Depressions, at 6 h after treatment. **(C)** Bulge on the surface of hemocyte, at 6 h after treatment. **(D)** Normal hemocytes, at 6 h after treatment. **(E)** Piercings, at 24 h after treatment. **(F)** Depressions, at 24 h after treatment. **(G)** Extracellular cell, at 24 h after treatment. **(H)** Normal hemocytes, at 24 h after treatment.

**Figure 12:**
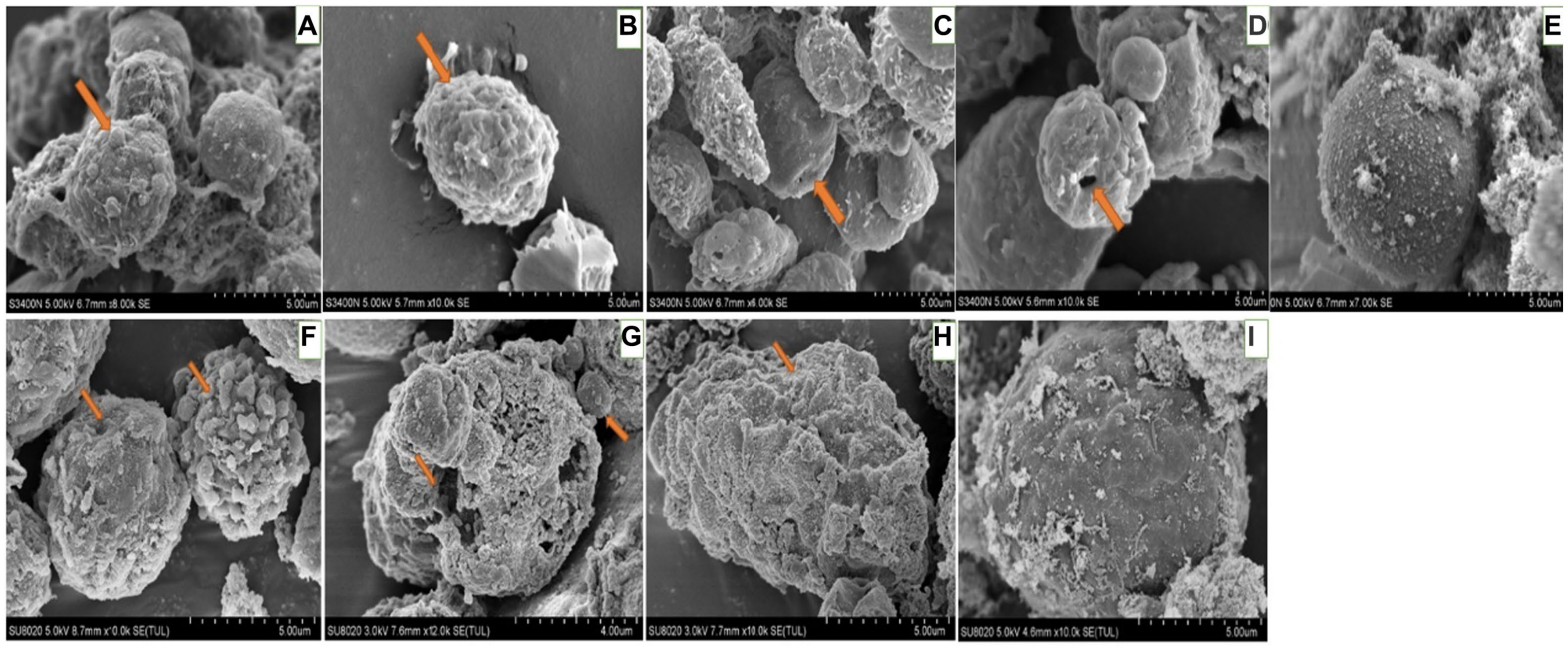
Scanning electron microscopic observation of the hemocytes of *Bombyx Mori* larvae treated with 0.1 μg/g DA. **(A)** Extracellular cell, at 6 h after treatment. **(B)** Shrinking, at 6 h after treatment. **(C)** Depression, at 6 h after treatment. **(D)** Perforation, at 6 h after treatment. **(E)** Normal hemocytes, at 6 h after treatment. **(F)** Extracellular cell and depression, at 24 h after treatment. **(G)** Shrinkage, at 24 h after treatment. **(H)** Perforation, at 24 h after treatment. **(I)** Normal hemocytes, at 24 h after treatment.

For hemocytes, treatment with DA at 0.01 μg/g for 6 h resulted in cells that were sunken, perforated, and protruding on the surface (Figure 11). At 24 h after treatment, the proportion of hemocytes with depressions and perforations was increased, and the extracellular cell was appeared. When larvae were treated with DA at 0.10 μg/g for 6 h, the effect was similar to that of the 0.01 μg/g dose; however, after 24 h, the number of hemocytes that showed depression, shrinkage, perforation, and puffing was increased. The extracellular cells were different from those treated with 0.01 μg/g of DA, and vesicles appeared on the cell surface, as shown in Figure 12.

**Figure 13:**
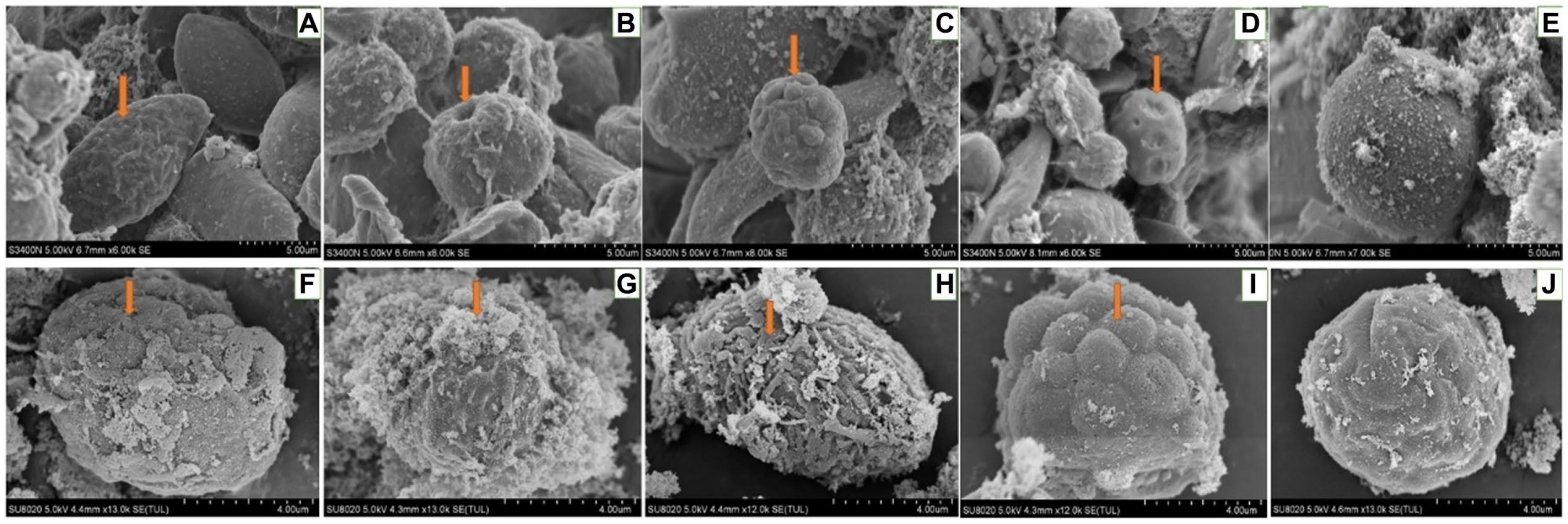
Scanning electron microscopic observation of the hemocytes of *Bombyx Mori* larvae treated with 1.0 μg/g of DA. **(A)** Shrinkage, at 6 h after treatment. **(B)** Perforation, at 6 h after treatment. **(C)** Foaming, at 6 h after treatment. **(D)** Depression, at 6 h after treatment. **(E)** Normal hemocytes, at 6 h after treatment. **(F)** Depression, at 24 h after treatment. **(G)** Cell rupture, at 24 h after treatment. **(H)** Shrinkage, at 24 h after treatment. **(I)** Bulge on the surface of hemocyte, at 24 h after treatment. **(J)** Normal hemocytes, at 24 h after treatment.

When larvae were treated with DA at 1.0 μg/g for 6 h, the effects were the same as those observed with the 0.01 and 0.1 μg/g doses. At 24 h after treatment, the hemocytes were lysed and releasing their contents (Figure 13).

### 3.5. Comparative effects of DA on tissues and organs

When larvae were treated with DA at higher concentrations (4.0 or 2.0 μg/g), the muscle cells were enlarged and the fat body was vacuolated. At 12 h after treatment with 4.0 μg/g of DA, the walls of Malpighian tubules became thinner. With DA treatments at lower concentrations (0.10 and 0.01 μg/g), the hemocyte structure was disrupted at 6 h, consisting of depressions, perforations, bulges on the cell surface, foaming and shrinking. In contrast, the muscle cells, fat body, and Malpighian tubules remained unaffected. At 24 h, the number of damaged hemocytes was increased, the fat body lysosomes were increased, the lipid droplets were deformed or incomplete, but the Malpighian tubules remained unchanged. With the rotenone and chlorpyrifos treatments, the fat body responded more quickly, with vacuolation observed at 30 min after treatment. Hence, hemocytes showed more sensitivity to DA than to the other two chemicals (Supplementary Table 1).

## 4. Discussion

Our data indicated that significant morphological changes were induced in *B. mori* larvae following injections with DA at different doses, that may be associated with binding sites of different proteins. Previous studies have shown that DA can bind to multiple proteins, suggesting that mode of action may differ depending on the target tissues and organs. At the higher dose rates, it is speculated that DA acts on multitarget sites of proteins that may damage the tissue and organs of the pest to a greater extent. Previous studies reported that DA can bind to BmPPI, one of the immunophilins, and to BmHSCP, a heat shock protein, to change their expression levels at a concentration of 88.1 μM (Zhang et al., 2017; Wang et al., 2019). In *B. mori* Bm12 cells, the protein BmTudor-sn was a target for DA binding at 100 μM, leading to a series of reactions (Wang et al., 2019). At concentrations from 10 to 50 μg/mL, DA binding to tRNA synthetases can disrupt protein synthesis (Wang et al., 2021). Previous studies reported morphological modifications resulting from DA binding to other proteins, including BmCRT, BmDPP3, and BmPDIA5 (Yin et al., 2022), and potentially the transmembrane protein 214 and the transport protein (Yin et al., 2021). In this study, the changes observed in different tissues and organs varied depending on the concentration of DA and the treatment time. It is speculated that when insects defend against DA, their hemocytes initiate the immune response against DA, which is followed by detoxification of DA by the fat body and Malpighian tubes. Our results are consistent with previous studies that DA is a small molecule with multiple targets that can act on different issues, such as muscles, Malpighian tubules and so on.

The hemolymph immunity is the main defense system to prevent small molecules or pathogens from damaging insects, and plays an important role in the process of defending against external invasion. When the *M. anisopliae* is infecting insects, it first attaches to the insect epidermis and forms an appressorium to penetrate the body wall to enter the abdomen, then it forms the hyphal body and spreads to the whole abdomen, followed by the secretion of secondary metabolites (including DA) to resist the cellular and humoral immunity in the insect abdomen. It is speculated that DA is more sensitive to hemolymph proteins than those in other tissues (Wang and Wang, 2017). DA can change the morphology and cytoskeleton of plasmatocytes, and inhibit the adhesion and diffusion, which can lead to a decrease in plasmatocyte phagocytic activity and the inhibition of encapsulation (Vilcinskas et al., 1997; Ríos-Moreno et al., 2017). The toxicity of DA against insect hemocytes was higher than that of other cells. At a low dose (i.e., 10 μL of 12.5 μg/mL), DA could induce obvious morphological alterations on hemocytes (Fan et al., 2013). When DA was acting on hemocytes of *B. mori*, a series of genes and proteins related to cell structure, metabolism, and immunity were significantly up-regulated or down-regulated. DA can coordinate with transcription factors *BmRel* and *BmRelish* to regulate the expression of immunity-related genes including antimicrobial peptides (Gong et al., 2014; Hu et al., 2017).

In this study, when the larvae of *B. mori* were treated with DA, the tissues and organs were found to have morphological modifications on blood cells such as depressions, perforations, and extracellular cells when DA dosage was low (0.01 μg/g), while the fat body and Malpighian tubules were not affected. When the larvae of *B. mori* were treated with a higher dosage of DA, the number of hemocytes increased, cells were shrinking, vacuolization of fat body appeared, the number of lysosomes increased, and the wall of Malpighian tubules became thinner. These results indicated that hemocytes are the most sensitive tissue to DA.

In conclusion, the tissues and organs of larval *B. mori* responded differently to different DA concentrations and treatment time, with the hemocytes observed to be the most sensitive target cells. The results provide substantial evidence that DA is an immunosuppressant by damaging the hemocytes, and it can play an important role in the development of mycopesticides and novel immunosuppressants for pest management.

## Data availability statement

The original contributions presented in the study are included in the article/Supplementary material, further inquiries can be directed to the corresponding author.

## Author contributions

FY and LH: methodology and validation. FY and QH: conduct the paraffin section observation and funding acquisition. FY, QH, ZL, XY, and PK: manuscript writing and editing. All authors contributed to the article and approved the submitted version.

## Funding

This research was supported by the National Natural Science Foundation of China (U1901205), Natural Science Foundation of Guangdong Province (2022A1515012395), Discipline Team Building Projects of Guangdong Academy of Agricultural Sciences in the 14th Five-Year Period (202105TD).

## Conflict of interest

The authors declare that the research was conducted in the absence of any commercial or financial relationships that could be construed as a potential conflict of interest.

## Publisher’s note

All claims expressed in this article are solely those of the authors and do not necessarily represent those of their affiliated organizations, or those of the publisher, the editors and the reviewers. Any product that may be evaluated in this article, or claim that may be made by its manufacturer, is not guaranteed or endorsed by the publisher.
