# Supplementary material for: Effects of destruxin A on hemocytes of the domestic silkworm, Bombyx mori
Source: Front Microbiol. 2023 Jun 2;14:1210647. doi: 10.3389/fmicb.2023.1210647 (PMC10272401; doi:10.3389/fmicb.2023.1210647)
Supplement: Supplementary file 1 [file Table_1.docx]

Supplementary Material

Effects of Destruxin A on Hemocytes of the Domestic Silkworm, *Bombyx mori*

Fei Yin*, Lina Hu, Zhenyu Li, Xiangbing Yang, Paul E. Kendra, Qiongbo Hu

*** Correspondence:** Qiongbo Hu: hqbscau@scau.edu.cn

| Chemical | Dose | Morphological response of treatment time (h) on organ/tissue of *B. mori* | | | | | |
| --- | --- | --- | --- | --- | --- | --- | --- |
|  | (μg/g) | Muscle cell | Fat body | Fat body | Malpighian tubules | Hemocytes | Hemocytes |
|  |  | 0.5-24 h | 0.5-12 h | 24-48 h | 12-48 h | 6 h | 24 h |
| DA | 0.01 | Normal | Normal | Increased of lysosome at 24 h | Normal | Depression, perforation, protuberance on the hemocyte | Depression, perforation cells, foaming |
|  | 0.1 | Normal | Normal | Increased of lysosome at 24 h | Normal | Sunken, perforated, shrinkage, protruding on the surface | Depression, shrinkage, piercing, puffing |
|  | 1.0 | Normal | Normal | Fat droplets became incomplete or split at 24 h | Normal | Depression, perforation, bulges, shrinkage | Depression, perforation, bulges, shrinkage |
|  | 2.0 | Enlarged in 1 h | Vacuolated at 2 h | More vacuolated | Normal | - | - |
|  | 4.0 | Enlarged at 1 h & elongated at 2 h | Vacuolated at 1 h | Vacuolated | Tube wall became thinner at 12 h | - | - |
| Chlorpyrifos | 0.1 | Normal | Vacuolated at 0.5 h | The vacuole ratio decreased | Normal | - | - |
|  | 1.0 | Elongated and enlarged at 1 h | Vacuolated at 4 h | Vacuolation intensified | Normal | - | - |
| Rotenone | 0.1 | Elongation and enlarged at 24 h | Vacuolated at 0.5 h | Vacuolation at 1 and 24 h | Tube wall became thinner at 48 h | - | - |
|  | 4.0 | Enlarged at 1 h | Vacuolated at 12 h | Vacuolation | Normal | - | - |
